# The Role of Immunometabolism in the Pathogenesis of Systemic Lupus Erythematosus

**DOI:** 10.3389/fimmu.2021.806560

**Published:** 2022-01-26

**Authors:** George Anthony Robinson, Meredyth G. Ll. Wilkinson, Chris Wincup

**Affiliations:** ^1^ Department of Rheumatology, Division of Medicine, University College London, London, United Kingdom; ^2^ Centre for Adolescent Rheumatology Versus Arthritis at University College London (UCL), University College London Hospital (UCLH) and Great Ormond Street Hospital (GOSH), University College London, London, United Kingdom; ^3^ Department of Rheumatology, University College London Great Ormond Street Institute of Child Health, Infection, Immunity and Inflammation Research and Teaching Department, University College London, London, United Kingdom

**Keywords:** systemic lupus erythematosus (SLE), immunometabolism, mitochondria, lipid metabolism, T cell, B cell, monocyte, autoimmunity

## Abstract

Systemic lupus erythematosus (SLE) is a chronic autoimmune disorder in which pathogenic abnormalities within both the innate and adaptive immune response have been described. In order to activated, proliferate and maintain this immunological response a drastic upregulation in energy metabolism is required. Recently, a greater understanding of these changes in cellular bioenergetics have provided new insight into the links between immune response and the pathogenesis of a number of diseases, ranging from cancer to diabetes and multiple sclerosis. In this review, we highlight the latest understanding of the role of immunometabolism in SLE with particular focus on the role of abnormal mitochondrial function, lipid metabolism, and mTOR signaling in the immunological phenomenon observed in the SLE. We also consider what implications this has for future therapeutic options in the management of the disease in future.

## 1 Introduction

Systemic lupus erythematosus (SLE) is a chronic autoimmune disorder characterized by the formation of autoantibodies directed against nuclear components. Clinically it may present with a wide array of manifestations and a variety of immunological phenomenon. In spite of recent advances in the management of the disease, therapeutic options remain limited and are often untargeted (1).

**Figure 1:**
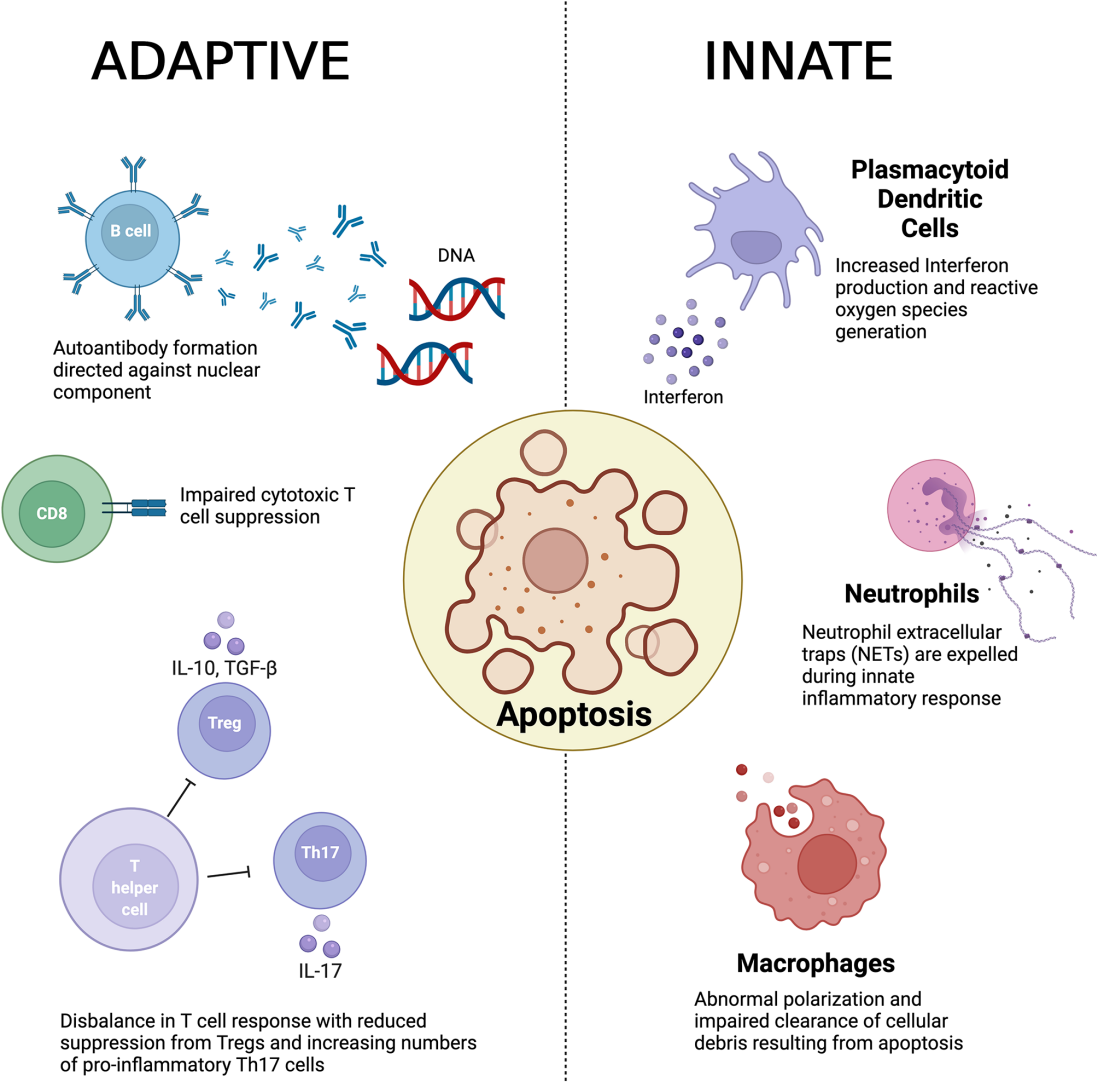
A summary of the key immunological abnormalities described in the pathogenesis of systemic lupus erythematosus.

The underlying pathogenesis of the disease is poorly understood although abnormal innate and adaptive immune responses have been implicated (2) and is summarized in **Figure 1**. Observed pathogenic innate responses include dysfunction of macrophages that appear to be defective in removing apoptotic material. It has been suggested that a result of this impaired clearance induces antigenicity to exposed cellular debris including nuclear components (3, 4). Macrophages (and their precursors, monocytes) have also been noted to display abnormal polarization in both animal models and in patients with SLE (5, 6). Abnormal neutrophil function has also been observed in the pathogenesis of SLE (7), with recent evidence also implicating the production of neutrophil extracellular traps (NETs) disease development (8, 9). In addition, plasmacytoid dendritic cells (pDCs) have been identified as another key innate immune driver that has been shown to play a key role in the production in interferon (INF) and generate reactive oxygen species (ROS) (10, 11).

The defective clearance of apoptotic matter by dysfunction in the innate immune response is believed to result in a loss of self-tolerance and in turn culminates in auto-antibody formation by B cells, which have been noted to show abnormal activation as well as aberrant expression. In turn this results in generation of anti-nuclear antibodies (ANA) and anti-double stranded DNA (anti-dsDNA) antibodies (4), a hallmark of the disease (12). Furthermore, B cells play a vital role in the development of immune complexes that contain self-antigen, which are deposited within various tissue. The resultant engagement of the Fc receptor and activation of the complement cascade in turn promotes inflammation (12, 13).

T cells also play a central role in the adaptive immune response and a number of abnormalities have been observed in the pathogenesis of SLE in both propagation and maintenance of the immune response. Regulatory T cells (Tregs) play a vital role in maintaining immune homeostasis in health through suppressing a hyperactive immune response. In SLE, an imbalance between pro-inflammatory T helper 17 (Th17) cells and Tregs has been demonstrated as a key contributor to the loss of self-tolerance (14, 15). Double negative T cells derived from patients with SLE have been shown to be an important producer of Interleukin(IL)-17 (16), whilst CD8^+^ cytotoxic T cells also demonstrate impaired suppressive function in SLE (17, 18).

The precise mechanism through which this occurs is not known, however, it is felt to involve a combination of genetic factors (19) and environmental triggers (including ultraviolet radiation and possible virus exposure) (20). In addition, given that the disease has a marked female predominance (9:1) there is a growing appreciation of the influence of sex hormones on the autoimmune responses observed (21).

More recently, abnormalities in immunometabolism have been detailed in the defective immune response seen in a variety of disease states including malignancy (22), diabetes (23, 24) and multiple sclerosis (25). This has shed new light on the way in which interactions between immunological and metabolic processes may induce the disease state. Immunometabolism also presents a variety of novel therapeutic targets for treatment in the future. In this review, we highlight the latest knowledge in the field of immunometabolism in SLE and describe how this may in turn translate into future clinical care.

## 2 Energy Metabolism

Immune cell activation and proliferation requires significant upregulation in terms of energy metabolism in order to induce and maintain the immunological response. Energy metabolism is dependent on two key pathways to generate adenosine triphosphate (ATP); glycolysis and oxidative phosphorylation (OXPHOS). In health, glycolytic pathways convert glucose to pyruvate and hydrogen ions that are essential for ATP synthesis. In comparison to glycolysis, OXPHOS occurs at the site of the electron transport chain (ETC) on the inner mitochondrial membrane.

## 3 Mitochondrial Dysfunction in SLE

Mitochondria are double membrane-bound organelles that generate cellular in the form of ATP, as well as regulating apoptosis. They cannot be replicated by the cell but are formed by binary fission. Each mitochondrion contains a set of circular genome that encode for RNA and proteins which are essential for mitochondrial oxidative phosphorylation. Here we explore the role of mitochondrial dysfunction in the immunopathogenesis of SLE.

### 3.1 B Cells (Auto-Antibodies)

The release of mitochondrial DNA (mtDNA) is a noted marker of acute and chronic disease (26, 27). MtDNA activates the innate immune system and can be a target for SLE associated autoantibodies. To identify mitochondrial autoantibodies, a study of 86 SLE patients and 30 healthy controls determined the occurrence of AmtRNA-IgG and Amt-IgM by quantitative ELISA. Both mtRNA immunoglobulins were significantly increased in the SLE patients (p=0.0002 and p=0.0493, respectively) (28). Antimitochondrial-M2 antibodies (AMA-M2) are associated with Primary biliary cirrhosis (PBC) and have been detected at increased levels in subacute cutaneous lupus erythematosus (SCLE) patients (29). In a study of 204 SLE patients, plasma samples were analyzed by ELISA for levels of anti-wMITO. Increased levels correlated to measures of disease activity SLEDAI-2K (p<0.0001) and SLAM (p=0.006), anti-dsDNA (p<0.0001) and other clinical measures (30). The presence of mitochondrial autoantibodies supports the role of mitochondrial damage in the pathology of SLE. Abnormal mitochondrial function in B cells derived from patients with SLE has more recently been identified. A study of 41 SLE patients and 29 healthy controls found that B cells derived from patients with lupus showed enhanced mitochondrial membrane hyperpolarization, suggesting that these cells are primed for activation. Furthermore, the degree of hyperpolarization correlated with SLEDAI-2K. The authors also noted that glutaminolysis, which generates essential metabolites for OXPHOS, played a key role in the differentiation into plasmablasts (31).

### 3.2 T Cells

T cell dysfunction in SLE could be attributed to mitochondrial hyperpolarization, reactive oxygen intermediates and reduced levels of ATP (32). Previous studies have demonstrated that T cells are dependent upon glycolytic energy production for the induction of the inflammatory effector response. However, mitochondrial metabolism has also been implicated in the more chronic activation of T cells observed in SLE (33). There is also evidence that in SLE, T cells have increased mitochondrial mass and size both due to defective mitophagy and increased biogenesis (34). Mitochondria contain a reservoir of Ca^2+^ ions. Increased mitochondrial mass and membrane potential (↑Δψ_m_) in SLE T-cells can increase intracytosolic Ca^2+^ fluxing when stimulated, in rapamycin treated SLE this was regulated (35). SLE but not healthy control T cells undergo necrosis after CD3/CD28 stimulation due to chronic mitochondrial hyperpolarization (MHP) (36). SLE T cell necrosis can also be caused by increased production of ROS and ATP depletion. Necrotic debris can induce a pro-inflammatory interferon response in plasmacytoid dendritic cells (pDCs) (37). Nitric oxide is released by monocytes which is a driver of MHP. In turn, T cells express intrinsic nitric oxide synthase (iNOS). A meta-analysis showed that there is higher expression of iNOS at both the mRNA and protein level (38). In T cells there is an increased response to IL-15 which in turn could contribute to increased mitochondrial biogenesis, though further studies need to be conducted to establish the role of cytokines in mitochondrial dysfunction (39). The status of T cell metabolic programming can be determined by mitochondrial remodeling as a signaling mechanism. This remodeling can change mitochondrial fusion to fission and equally oxidative phosphorylation to aerobic glycolysis. These mechanisms are distinct between effector and memory T cells (40). In SLE T cell oxidative stress is pronounced, increased expression of the mitochondrial protein genes VDAC1 and SOD2 are associated with an increase in mitochondrial mass and oxidative stress (36, 41). Other genes associated with mitochondrial dysfunction in SLE are ESRRG, a mitochondrial metabolism regulator, and UCP2, involved in ROS generation and ATP production (42, 43). It has been shown that due to oxidative stress, surface glycoprotein CD3ζ chain is damaged and replaced by FcϵRIγ chain in SLE T cells. The TCR/CD3/FcϵRIγ complex is up-regulated in effector T-cells and has been shown to be increased in SLE T cells (44).

More recently there is growing evidence to suggest that targeting T cell metabolism may be a potential therapeutic target for the management of SLE in the future. N-acetylcysteine (NAC) is used clinically as an anti-oxidant therapy and could have a role in targeting oxidative stress in SLE. In a randomized, double-blind, placebo trial of NAC in 36 SLE patients there was significant clinical improvement on 2.4 g and 4.8 g dose in terms of Systemic Lupus Erythematosus Disease Activity Index 2000 (SLEDAI-2K) at 1 months (p=0.0007), 2 months (p=0.0009), 3 months (p=0.003) and 4 months (p=0.0046). This study showed that NAC successfully blocks the mammalian target of rapamycin (mTOR) in T cells (45). Combination treatment with Metformin (a mitochondrial metabolism inhibitor, more commonly used in the treatment of diabetes) and 2-deoxy-d-glucose (2DG) in lupus prone mice has also shown promise, resulting in reduced INF-γ production. In addition, mice treated with this therapy showed a reversal of the disease process and reduction in both anti-dsDNA and ANA titers (33). Furthermore, targeting T cell glycolysis has also been demonstrated to specifically reduce the production of follicular helper T (T_FH_) cells, which have been implicated in the pathogenesis of SLE (46). Glycolysis has also been investigated as a therapeutic target in another subtype of CD4^+^ T cells, Th17 cells that predominantly use glycolysis for energy metabolism. In a study of T cell derived patients with SLE, it was found that by blocking pyruvate dehydrogenase phosphatase catalytic subunit 2 (PDP2), a vital enzyme in the glycolytic pathway, it was possible to limit Th17 differentiation (47). Inhibition of glutaminolysis (a key source of energy for effector T cells) has also been shown to impact on glycolytic pathways and result in a similar reduction in Th17 differentiation in samples derived from both patients and lupus prone mice, thus suggesting this could also be a potential metabolic therapeutic target in the future Furthermore, the authors also found that inhibition of glutaminolysis reduced Th17 Hypoxia Inducible Factor (HIF)-1α levels, which plays a central role in Th17 development (48). These studies suggest that through augmentation of T cell metabolic pathways it may be possible impair abnormal T cell cytokine production and differentiation.

### 3.3 Neutrophils

Neutrophils taken from SLE patients and healthy control INF primed neutrophils extrude high levels of oxidized mitochondrial nucleoids that act as potent interferogenic complexes, this affect could be due to failed mitophagy. TFAM enables neutrophil-derived mtDNA to be internalized and in turn can become a potent pDC activator. INF/αRNP can divert extruded oxidized mtDNA into lysosomes. In turn this drives the formation of ox mtDNA/TFAM complexes which then accumulate in the cytosol and the mitochondria itself. In SLE there are high levels of these oxidized nucleoids in the blood and the neutrophils themselves. In addition, autoantibodies against oxidized mtDNA are present in some SLE patients, proposing ox mtDNA as an autoantigen (49). In SLE and juvenile dermatomyositis (JDM) there are increased levels of neutrophil extracellular traps (NETs) and these have been found to contain mtDNA (50). In SLE, mitochondrial ROS are necessary for NETosis of low density granulocytes (8). Inhibiting mtROS may reduce the INF response in these diseases. In SLE, neutrophils are key to activating the inflammatory mechanism of mtDNA.

### 3.4 Monocytes

A complex study of SLE monocytes showed that excessive INFα in SLE damaged mitochondrial respiration. In the monocytes, SLE compared to healthy control, the results showed increased mitochondrial membrane potential (p<0.0005), PINK1 mRNA (p<0.005), mtDNA content (p<0.005) and JC1 aggregates (p<0.05). These results were re-produced when healthy donor monocytes were cultured with INFα for 18hrs (51). This delineates the cyclical relationship of INFα with mitochondrial dysfunction.

Across the innate and adaptive immune cells there is strong evidence that mitochondrial dysfunction plays an important role in SLE immunopathogenesis. Therefore, is an important therapeutic target to consider.

## 4 Abnormal mTOR Signaling in SLE

Another important group of substrates involved immune cell metabolism are proteins, peptides and amino acids. There is now a growing appreciation of their role in autoimmunity, in particular in relation to their effects on T cell differentiation and function. This relies upon the activation of the serine-theonine protein kinase, mTOR, which exists in two separate complexes known as mTORC1 and mTORC2 (52). Furthermore, mTOR is essential in the maintenance of immune cell homeostasis through its roles in inducing metabolic signals that in turn drive cell growth, activation, proliferation and survival (52–55).

In health, mTORC1 plays a key role in the suppressive function of Tregs, a mechanism that has been demonstrated to be abnormal in many autoimmune conditions (55–57). In SLE, abnormalities within mTOR pathways have been shown to induce immune cell differentiation and proliferation, secretion of pro-inflammatory cytokines and increased ROS production (58). Previous studies have demonstrated the role of mTORC1 activation in CD4^+^ T cells derived from patients with SLE (53) and has suggested that this may be due to mitochondrial dysfunction (45). More specifically, mTOR abnormalities have been reported to alter the balance between Th17 T cells and Tregs to the extent that it promotes a state autoimmunity (59). This increase in mTORC1 activity has been demonstrated following increased glycolysis and also associates with reduced levels of autophagy (54), which is impaired in the pathogenesis of SLE (60).

There is growing evidence that targeting mTOR may also be an effective treatment in the management of SLE clinically. Sirolimus (Rapamycin), an immunosuppressive agent used in preventing graft rejection in solid organ transplantation and known mTORC1 inhibitor, has already been studies as a potential treatment for SLE. Inhibition of mTOR with Rapamycin has already been shown to reduced INF production by monocytes derived from patients with SLE *in vitro* (61). In a previous open-label study in 43 patients with active SLE found that following 12 months of treatment with Sirolimus, disease activity was significantly reduced, and concurrent steroid dose was also significantly lower following one year of treatment. Immunologically, Sirolimus was also noted to induce increased numbers of Tregs, which suggests a recovery immune homeostasis with the treatment. Furthermore, T cell produced IL-4 and IL-17 levels were also significantly lower following treatment (62). Although there is a lack of large randomized, placebo-controlled trials of the drug in lupus, a recent meta-analysis of nine studies containing a total of 145 patients concluded that Sirolimus showed promise as a treatment option. It was suggested that the drug was well tolerated (although hematological and mucocutaneous adverse events were the most frequently reported) (63). Inhibition of mTOR with Sirolimus additionally was associated with higher rates of dyslipidemia, which is important given the growing evidence for abnormalities in lipid metabolism in SLE.

## 5 Lipid Metabolism in SLE

The metabolism of lipids is a fundamental process used by immune cells for different energy demands, cell signaling and function. Lipids serve as precursors for bioactive metabolites and components of cellular membranes, which have both direct and indirect regulatory implications for signal transduction, gene regulation and cellular activation. Immune cell subsets have different metabolic demands for lipids, such as mitochondrial beta-oxidation of lipids for anti-inflammatory functions in regulatory T cells, against a higher dependency on glycolytic pathways for growth and proliferation in effector T cells (64). Dysregulated lipid metabolism has been heavily implicated in SLE at both the systemic and cellular level and both have been described in the context of cardiovascular comorbidities.

### 5.1 Lipid Metabolism and Cardiovascular Disease in SLE

Patients with SLE have an increased risk of developing cardiovascular disease (CVD) beyond traditional risk factors and CVD is a leading cause of mortality for patients (65). This CVD risk is largely due to dyslipidemia (altered lipid metabolism), a common feature of SLE (66). Dyslipidemia can accelerate atherosclerosis, the lipid build-up and chronic inflammation of the large arteries (67). This involves an imbalance between atherogenic low and very low density lipoproteins (LDL and VLDL), and atheroprotective high density lipoproteins (HDL) known to transport lipids too and away from atherosclerotic plaques respectively. Dyslipidemia in SLE includes both elevated LDL and reduced HDL (66, 68–70) which, along with chronic inflammation, accelerates atherosclerotic processes.

### 5.2 Lipid Metabolism in Immune Cell Function in SLE

Lipoprotein metabolism can also influence immune cell function and inflammation in SLE (71). It is well established that innate immune cells, including macrophages, take up oxidized (ox)LDL particles *via* scavenger receptors in atherosclerotic plaques, leading to lipid saturation, pro-inflammatory cytokine production, and recruitment of other inflammatory cells (72). This process could be exacerbated in SLE due to the increased circulating levels of LDL, thus, increasing atherosclerosis progression. In addition, macrophage function is likely to be altered *via* direct lipid activation of the nuclear liver-X-receptors (LXRs), which regulate cellular cholesterol levels and immune functions through transcriptional changes, such as those involved in IL-23 and IL-17 production and phagocytic pathways (73). The direct effect of a hyperlipidemic environment on the T cell inflammatory profile in SLE has also been investigated (74, 75) and oxLDL has been shown to increase T cell activation indirectly through monocyte uptake (76). T cells are key for the adaptive immune system and upon activation, T cells proliferate, migrate to inflamed sites, such as atherosclerotic plaques, and acquire functions that mediate the immune response (77). The T cell plasma membrane (PM) is made up abundantly of lipids, such as cholesterol and phospholipids, and proteins, both of which are essential to facilitate cellular signaling for inflammatory outcomes such as cytokine production and proliferation (78). Patients with SLE and other autoimmune diseases have altered T cell membrane cholesterol and glycosphingolipid levels (79, 80). This alters the composition of signaling platforms called lipid rafts, where T cell receptors provide stimulatory signals to control cellular function and inflammation (81, 82). This is partly due differences in the expression of genes responsible for lipid metabolism in SLE (71), however, this could also be due altered cellular altered uptake of cholesterol from LDL/VLDL and efflux of cholesterol to HDL; this process has been speculated in pathogenic mechanisms of multiple sclerosis (83). Altered lipid rafts have also been described in the context of dysfunctional B cell signaling in SLE (84). Altered lymphocyte function through dyslipidemia in SLE is also likely to be mediated through LXRs (82, 85, 86). Together, research strongly suggest that lipid metabolism could be targeted therapeutically to control cellular functions and inflammation, highlighting the need for a better use of lipid modification strategies in SLE.

### 5.3 Lipid Metabolism as a Therapeutic Target

Some conventional therapeutics currently being used to treat SLE have shown beneficial effects on lipids, including hydroxychloroquine on LDL lowering (87). Despite this, deaths associated with cardiovascular comorbidities are still high (65, 88) suggesting that additional, more specific lipid modifying therapies are in demand for patients with SLE. Despite statin trials in SLE showing mixed results regarding cardiovascular outcome measures (89–91), therapeutically lowering circulating lipid levels has been shown to improve autoimmune disease symptoms (92) and using these therapies to directly modify lipid rafts *in vitro* has also been shown to normalize signaling in T cells from SLE patients (80, 93).

Taken together, differences in lipid metabolism in patients with SLE contributes to disease pathogenesis, inflammation and CVD risk through atherosclerosis. Therapeutic intervention with lipid modifying drugs already approved for use worldwide, such as statins, could be promising strategies to control atherosclerosis and inflammation in SLE. The success of future clinical trials and the therapeutic application of these treatments is likely to be dependent on correct patient stratification. Reducing CVD risk in SLE patents from a young age will be a huge breakthrough for long term patient outcomes and quality of life.

## 6 Conclusions

**Figure 2:**
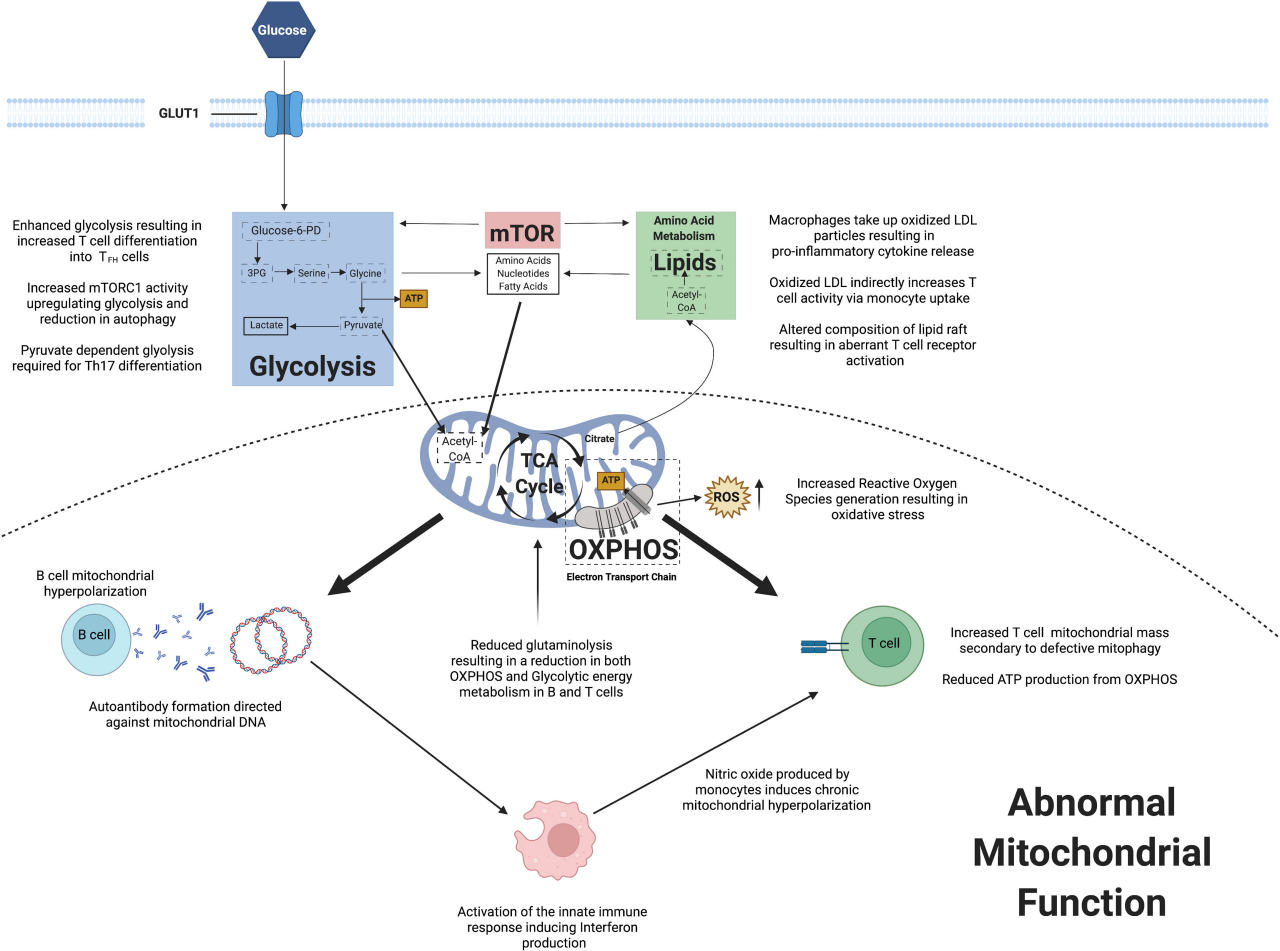
A summary of observed changes in cellular metabolism reported in systemic lupus erythematosus.

The field of immunometabolism has enhanced our understanding of the key changes in cellular homeostasis and how this can result in autoimmune conditions including SLE. Observed mitochondrial dysfunction has implications for immune cell energy metabolism and also ROS generation. Abnormalities within mTOR signaling may induce promote immune cell differentiation and proliferation, whilst also stimulating pro-inflammatory cytokine production. Lipid metabolism has been shown to potentially play a role in immune cell signaling. **Figure 2** summarizes the key changes in immunometabolism observed in SLE to date.

In conclusion, our understanding of immunometabolism in SLE is rapidly increasing and main soon translate to newer agents being developed specifically to restore immune cell homeostasis in the disease.

## Author Contributions

MW was responsible for writing Section 3 (Mitochondrial dysfunction in SLE). GR was responsible for writing Section 4 (Lipid metabolism in SLE). CW was responsible for the remaining sections and editing the final manuscript. All authors contributed to the article and approved the submitted version.

## Funding

GR is funded by a combined Lupus UK and the Rosetrees Trust grant (ref M409). MW is funded by Cure JM and GOS BRC. CW is funded by Versus Arthritis (ref 21992 and 22600) and Lupus UK.

## Conflict of Interest

The authors declare that the research was conducted in the absence of any commercial or financial relationships that could be construed as a potential conflict of interest.

## Publisher’s Note

All claims expressed in this article are solely those of the authors and do not necessarily represent those of their affiliated organizations, or those of the publisher, the editors and the reviewers. Any product that may be evaluated in this article, or claim that may be made by its manufacturer, is not guaranteed or endorsed by the publisher.
